# When Metabolomics Meets Quantitative Genetics: An Integrative Strategy to Elucidate Plant Resistance Mechanisms

**DOI:** 10.1111/pce.70328

**Published:** 2025-12-15

**Authors:** Romane Lapous, Komla Exonam Amegan, Bernard Caromel, Charles‐Eric Durel, Anne‐Violette Lavoir, Julie Ferreira de Carvalho, Romain Larbat

**Affiliations:** ^1^ Univ Angers, Institut Agro, INRAE, IRHS, SFR QUASAV Angers France; ^2^ Université de Lorraine, LAE, INRAE Nancy France; ^3^ INRAE, GAFL Montfavet France; ^4^ Université Côte D'Azur, INRAE, UMR ISA Nice France

**Keywords:** biotic interactions, genetic control, QTL co‐localisation, resistance durability, specialised metabolism

## Abstract

Gene pyramiding in crop varieties offers a promising strategy to achieve sustainable production and reduce reliance on pesticides. However, stacking resistance genes without understanding their biological functions may result in transient protection. Although numerous studies have mapped loci associated with resistance to biotic stresses, the underlying molecular mechanisms remain poorly characterised. Resistance genes are often involved in pest/pathogen recognition, whereas quantitative trait loci (QTLs) may act in other steps of plant immunity such as signalling and defence pathways. In parallel, specialised metabolites have attracted growing attention as key defence components, acting as antimicrobial or repellent agents. While both fields encounter challenges to precisely decipher plant defence mechanisms, making use of metabolomics on segregating populations could bypass some of these limitations. In this review, we introduce an approach based on the identification of metabolic QTLs within populations where resistance QTLs segregate, enabling the detection of genomic co‐localisations between both types of QTLs. This integrative framework can reveal specific metabolic signatures associated with resistance, thus refining hypotheses on the mode of action of resistance QTLs. Ultimately, elucidating the genetic architecture of specialised metabolism in relation to quantitative resistance will inform on more effective combinations of defence mechanisms for breeding resistant varieties.

## Summary statement

- This review presents an approach using metabolic QTL mapping in populations segregating for resistance QTLs in order to identify genomic co‐localisations.

- This strategy helps highlight specific candidate genes and metabolites and refine hypotheses on the mode of action of resistance QTLs

## 1 Introduction

Crops face numerous biotic threats from pests and pathogens, which can lead to yield loss of up to 40% (Savary et al. 2019). To mitigate these challenges, chemical pesticides are widely used despite their negative consequences on the environment and human health. To reduce their impact, complementary agronomic tools are currently studied and implemented as part of Integrated Pest Management strategies in agroecosystems (see Zhou et al. 2024). Among these levers, genetic resistance through introgression of favourable alleles remains a predominant and efficient way to propose genotypes adapted to their biotic environment.

Resistant cultivars have been widely deployed across various crops, helping to reduce yield loss and moderate dependence on chemical control. Notable examples include the *Xa* genes in rice, which have provided effective resistance against bacterial blight worldwide (Chen et al. 2021), and the *Dn4* gene in wheat, which confers protection against the Russian wheat aphid (*Diuraphis noxia*) (Smith et al. 2004). However, despite these remarkable examples, maintaining genetic resistance over time remains a significant challenge. Genetic resistance can erode rapidly due to pathogen adaptation, raising concerns about their durability (Brown 2015). Importantly, this breakdown frequently results from an incomplete understanding of the molecular mechanisms underlying resistance, particularly for quantitative resistance controlled by multiple genes.

Pyramiding resistance genes, by combining several sources of resistance, has long been proposed as a strategy to enhance the durability of genetic control (Parlevliet 2002). Yet, its benefits may be short‐lived, leading to increasing interest in diversifying resistance mechanisms within elite crops as a more sustainable approach (Li et al. 2001; Fry 2008; Delourme et al. 2014; Lasserre‐Zuber et al. 2018). Achieving this, however, remains challenging, as it requires the identification and strategic combination of complementary loci whose biological functions are often poorly understood, resulting in only transient resistance gains. Integrating mechanisms acting at different stages of pathogen recognition and plant defence is expected to increase the evolutionary cost for pests and pathogens, thereby reducing the risk of resistance breakdown (Pilet‐Nayel et al. 2017; Zhang et al. 2021).

While large effect resistance genes (or R genes) have been extensively cloned and functionally characterised, the molecular mechanisms underlying quantitative resistance remain poorly understood. Evidence shows the involvement of R genes in pest/pathogen recognition, while quantitative trait loci (QTLs) may act in other steps of plant immunity such as signalling and defence pathways. Importantly, specialised metabolites have emerged as key end‐players of plant defence, with many of them, exhibiting antimicrobial or insecticidal effect (Block et al. 2020; Taye and Borkataki 2020). However, establishing the causal link between genetic control and metabolic variation has proven challenging when using traditional approaches separately. To address these limitations, an approach combining quantitative genetics with metabolomics has emerged. This approach relies on the identification of metabolic QTL (mQTLs) in populations where resistance QTL (rQTLs) segregate, and on the determination of genomic co‐localisation between both types of QTLs. This integrative strategy offers a powerful means to connect genetic architecture with biochemical mechanisms, potentially revealing the metabolic basis of quantitative resistance. By identifying co‐localising mQTLs and rQTLs, researchers can narrow down candidate genes and metabolites simultaneously, providing mechanistic insights to inform effective gene pyramiding strategies.

In this review, we highlight this integrated mQTL/rQTL approach as a strategy to identify the molecular mechanisms implicated in plant‐pest and ‐pathogen interactions. This approach may help to better understand the variability and genetic control of defence‐related molecules and to inform breeders on the most efficient molecules and alleles to integrate into new resistant varieties. After a brief overview of the contributions of both quantitative genetics and metabolomics to understand plant resistance, we focus on studies demonstrating the power of combining genetic control of specialised metabolism with resistance traits through genomic co‐localisation. Finally, we discuss the advantages, limitations, and challenges of this strategy for developing more durable resistant varieties.

### 1.1 Genetics of Plant Resistance

1. **Identification of plant resistance genetic loci**
  For a long time, plant responses to pests and diseases have been strictly categorised as either ‘qualitative’ or ‘quantitative’, referring to distinct aspects of resistance at the genetic and phenotypic levels. In a given population of genetically different individuals, resistance phenotype may be described as either qualitative (often meaning ‘complete’) or quantitative (or ‘partial’) (Roux et al. 2014; Niks et al. 2015; Corwin and Kliebenstein 2017). However, these terms are also used to describe the genetic control of a trait: monogenic and polygenic inheritances are respectively defined as ‘qualitative’ (associated genes are referred to as ‘major’ or ‘R’ genes) and ‘quantitative’ (involving notably resistance Quantitative Trait Loci; rQTLs). Morever, quantitative phenotypes may have qualitative inheritance and vice versa (Niks et al. 2015). Furthermore, additional parameters, such as pest and pathogen diversity, environmental conditions, and natural mutations in genes, are believed to alter the strength of resistance conferred by R genes, leading to quantitative variation in what was previously termed ‘qualitative’ resistance (Roux et al. 2014). Therefore, combining resistance loci based on their genetic control or observed phenotype might not be the most accurate way to enhance resistance durability. Instead, it has been proposed to integrate complementary molecular pathways in breeding material.
  Hundreds of studies aiming at identifying genomic regions implicated in quantitative resistance to pests and diseases have been undertaken and mostly rely on two main strategies: linkage mapping and genome‐wide association study (GWAS). Both strategies involve genotyping of individuals with molecular markers and phenotyping for traits of interest, followed by statistical analyses linking markers to the trait variation. Estimation size of the identified genomic regions depends on the effect of the resistance gene, mapping populations available, which will then influence the extent of studied diversity, recombination profiles, marker density and most of all, the ability to identify causal genes associated with traits of interest (St. Clair 2010).
  Linkage mapping uses artificial crosses between genotypes to create mapping populations. Various population types can be developed according to the species biology and research goals which will impact result accuracy (see Box 1; reviewed in Collard et al. 2005; Xu et al. 2017). In addition, limited generations restrict the number of recombination events, leading frequently to large confidence intervals that span many genes. Extensive mapping resolution can be achieved through fine‐mapping studies implying a high number of offsprings (~500 to > 10 000 if available) which will increase the number of recombination events. Additionally, multi‐parental populations, such as MAGIC and NAM progenies (see Box 1), are suitable to overcome the limitations of using bi‐parental families by increasing the number of recombination events and allelic diversity (Korte and Farlow 2013; Gage et al. 2020; Scott et al. 2020).
  GWAS relies on diversified material (composed of elite cultivars, landraces, wild relatives and/or exotic accessions) with hundreds of genotypes providing a wide range of allele combinations (Xu et al. 2017; Demirjian et al. 2023). However, this strategy is less effective at detecting rare alleles with large effect sizes, which may be common for resistance genes underlying QTLs (Louthan and Kay 2011; Korte and Farlow 2013).
2. **Molecular mechanisms underlying resistance genetic loci**

Box 1 **Plant populations for genetic analyses: Definition and methods.**

**Autogamous species**

*Biparental*

**F1 progenies:** cross between two individuals with homozygous genomes leading to a generation of heterozygous individuals.

**F2 and Back‐Cross (BC)**: F2 progenies are derived from the selfing of a single F1 plant, while BC progenies result from crossing a F1 plant with one of its parents. Each individual of these two types of progenies is unique because different recombination events happened in each individual. Replicates are only possible by exploiting vegetative propagation, which is not a common mode of reproduction in most autogamous species.

**Recombinant Inbred Lines (RIL):** RIL are derived from self‐pollinated F2 plants over six to eight generations. One seed per plant is conserved at each generation, which is called ‘single‐seed selection’. Similarly to F2 progenies, RIL are composed of unique individuals but they are fully homozygous. They can be maintained indefinitely through selfing.

**Doubled Haploids (DH):** DH progenies are produced by doubling the chromosome number of gametes in F1 plants, with plants being regenerated using tissue culture techniques. They are equivalent to RIL in term of recombination event number. They can be maintained indefinitely through selfing.

**Near‐Isogenic Lines (NIL):** NIL individuals are nearly identical except for a chromosomal region, containing the locus of interest. They are created through successive backcrosses during which the locus of interest is selected, usually using molecular markers. In many cases, NIL are constructed to confirm previously identified genetic loci that explain part of the variation observed in a specific trait of interest.

**Introgression Lines (IL):** IL are developed by successive backcrossing of an interspecific F1 hybrid with the cultivated parent. The introgression of the region of interest is typically managed through marker‐assisted selection.

**Backcross Introgression Lines (BIL):** BIL are produced by two successive backcrosses of an interspecific F1 hybrid with the cultivated parent, followed by six generations of selfing. They are similar to RIL but one parent is usually a close‐related species. They can be maintained indefinitely through selfing.

*Multiparental*

**Nested Association Mapping (NAM):** NAM populations consist of numerous RIL progenies, resulting from the crossing of several donor parents with a common parent. Hundred of RIL can be derived from each biparental cross, leading to a higher number of individuals. This material increases allelic diversity and QTL resolution comparing to RIL studies.

**Multi‐Parent Advanced Generation Inter‐Cross (MAGIC):** MAGIC progenies are created by following a tunnel crossing pattern, generally starting with multiple parents (4, 8 or even 16). Parents are crossed and resulting F1 plants are then inter‐crossed over multiple generations. Thus, resulting genotypes are random mosaics of the founder ones.

**Alternatives for strict allogamous (i.e non self‐pollinating) species**

**Pseudo‐F1, ‐F2 and ‐BC:** Genomes of allogamous species are highly heterozygous thus F1 plants result from crossing two unrelated individuals, leading to pseudo‐F1 progenies. An additional generation, to produce pseudo‐F2 or pseudo‐BC, is sometimes carried out. Pseudo‐F2 progenies result from the cross of two full‐sibling pseudo‐F1 plants; and pseudo‐backcross progenies are produced by crossing one pseudo‐F1 plant with one of its parents or with another accession.

From such approaches, many sources of resistance have been mapped in various crops and are considered as valuable resources in breeding. On a functional aspect, accumulation of evidence has proposed the role of specific mechanisms behind R gene expression. From the Flor's model (1971) to the ‘zig‐zag model’ (Jones and Dangl 2006), R genes have always been associated with the recognition of threats in plants. This recognition might be ensured by direct or indirect protein interactions, through intra‐cellular or cell‐surface receptors, that target ‘specific virulence effectors’ or more conserved microbial‐, damage‐ or herbivore‐associated molecular patterns (respectively, MAMPs, DAMPs or HAMPs). To date, hundreds of R genes have been cloned and most of these genes were indeed described as encoding receptor proteins that recognise pathogens. Nonetheless, only a few of them led to a loss of plant susceptibility by disarming the pathogen and interrupting key pathogenic processes (Kourelis and van der Hoorn 2018; van der Burgh and Joosten 2019).

Regarding rQTLs, the underlying molecular mechanisms are much less studied compared to R genes. To our knowledge, only a few genes with partial effects behind rQTLs have been cloned (Niks et al. 2015; French et al. 2016; Pilet‐Nayel et al. 2017). Some of these genes have been shown to be involved in recognition as an altered or ‘weaker’ form of R genes (Li et al. 1999; Vollrath et al. 2021). In contrast, other rQTLs have been proposed to be involved in signalling or in downstream pathways. For instance, two rQTLs have been associated to kinase genes in *Triticum* and Arabidopsis, suggesting a role in signalling (Fu et al. 2009; Huard‐Chauveau et al. 2013). Another study in maize showed the association of a rQTL to three foliar diseases with a candidate gene encoding an enzyme involved in the phenylpropanoid pathway and lignin production (Yang et al. 2017). In potato, quantitative resistance to late blight was mainly associated with cell wall thickening due to deposition of hydroxycinnamic acid amides, flavonoids and alkaloids (Yogendra et al. 2014, 2015). To sum up, the causal genes underlying rQTLs encode kinases, transcription factors, metabolic enzymes, transporters and altered receptors, which cover various functions within the array of defence mechanisms (Pilet‐Nayel et al. 2017).

In recent decades, development of Next‐Generation Sequencing tools has enhanced genotyping capacities rendering phenotyping as the major bottleneck since quality and reproducibility of trait measurements are crucial to avoid biases and enhance the resolution of QTL mapping (Huang and Han 2014). Resistance to insects and pathogens is usually measured through visible/calculated traits reflecting the plant defence capabilities (e.g., necrosis, leaf spot, presence of defensive structures like trichomes), the plant damages (e.g., level of leaf and fruit damages) or the pest susceptibility (e.g., survival rate, oviposition rate, pathogen quantification). Assessing multiple traits for one pathosystem could lead to the detection of several QTLs that may co‐localise, offering additional clues to determine molecular mechanisms of resistance. However, scoring disease usually relies on ordinal or semi‐quantitative scales, which provide less precision than quantitative methods (Poland and Nelson 2011). Development of new phenotypic approaches is, therefore, crucial to get more insights into the biological processes involved in plant resistance. One promising direction is the study of endophenotypes (Te Pas et al. 2017)—such as RNA (transcriptomics), proteins (proteomics), and metabolites (metabolomics)—which can help researchers to accurately capture the subtle variations in plant responses to environmental cues. Metabolites are indeed promising as direct products of gene expression and potential effectors of resistance mechanisms. The following sections will focus on systematic approaches to characterise plant defence metabolites (Part II) and link them to genetic control (Part III).

### 1.2 Metabolomic Approaches and Plant Defence

**Figure 1:**
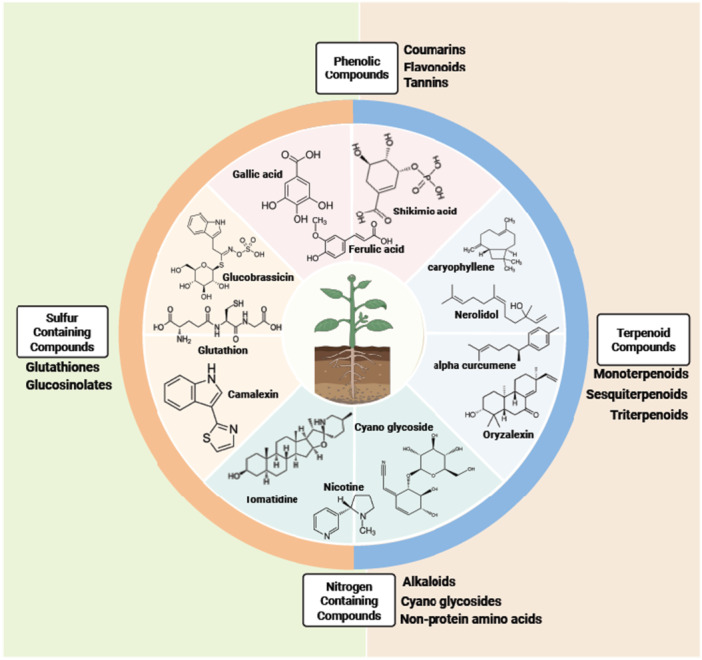
Representation of the structural diversity of the plant specialised metabolism, categorised into four families. Phenolic compounds (for reviews, see Dong and Lin 2021; Ramaroson et al. 2022) include flavonoids, hydroxycinnamic acid esters and amides, tannins and the precursors of lignin. Terpenes are hydrocarbons characterised by a number of isoprene units while terpenoids may have additional functional groups (for reviews, see Boncan et al. 2020; Nagegowda and Gupta 2020). Alkaloids are nitrogen‐containing compounds composed of a heterocyclic ring (for reviews, see Matsuura and Fett‐Neto 2015; Bhambhani et al. 2021). Finally, the fourth category of plant defence metabolites classically considered is the sulfur‐containing compound group including the well‐described glucosinolates specific to the Brassicales order (for reviews, see Bloem et al. 2005; Künstler et al. 2020; Mitreiter and Gigolashvili 2021).

1. **Summary of plant defence metabolites**
  Due to their sessile nature, plants have acquired the ability to produce and release a huge diversity of metabolites, estimated between 200 000 and 1 000 000 in the plant kingdom, with around 5000 per plant species (Dixon and Strack 2003; Afendi et al. 2012; Venegas‐Molina et al. 2021; Huang and Dudareva 2023). Plant metabolites are usually divided into two categories, namely (i) primary or common metabolites (organic, nucleic and amino acids, sugars, lipids) presented in all organisms and essential for the cell viability and (ii) secondary or specialised metabolites which are species‐specific and critical for the plant‐environment interactions (Firn and Jones 2009; Fàbregas and Fernie 2021). While defence metabolites belong mainly to this latter category, primary metabolites may also contribute to plant defence by providing energy, reducing power and elemental substrates for the synthesis of induced metabolites, and sometimes exhibiting direct activity against pest and pathogens (Bolton 2009; Zaynab et al. 2019).
  The main plant specialised metabolites involved in plant defence include terpenoids, phenolics, sulfur (S)‐ and nitrogen (N)‐containing compounds with specific structures (Figure 1) (Hartmann 2007; Jan et al. 2021; Huang and Dudareva 2023). Each of these metabolite families has been the subject of relevant reviews regarding their biochemistry, biosynthesis and bioactivities (Vogt 2010; Pichersky and Raguso 2018; Blažević et al. 2020; Lichman 2021; Mitreiter and Gigolashvili 2021). They could contribute to defence either directly by repelling, deterring or altering pest performance or pathogen life cycle, either indirectly by recruiting natural enemies of specific pests/pathogens (Chen et al. 2008; Dicke and Baldwin 2010; Erb et al. 2012; Maag et al. 2014; Aljbory and Chen 2018; Hammerbacher et al. 2019; Singh et al. 2021). The accumulation profile of plant defence metabolites is highly variable, either systemic or local, either constitutive (phytoanticipins) or induced by the pathogen/herbivore (phytoalexins). These differences are related to the plant species, the metabolite nature and several other factors such as plant phenology.
  Overall, understanding the role of specialised metabolism on plant defence is challenging for several reasons including the huge diversity of structure and usually low concentrations of these metabolites, the specificity of their distribution in the plant lineage, together with the specificity of their implication according to the pathosystem. These challenges highlight the need for systematic analytical approaches to characterise plant metabolomes and link metabolic diversity to defence functions.
2. **Strategies to identify biomarkers in plant‐pathogen and plant‐pest interactions**

**Figure 2:**
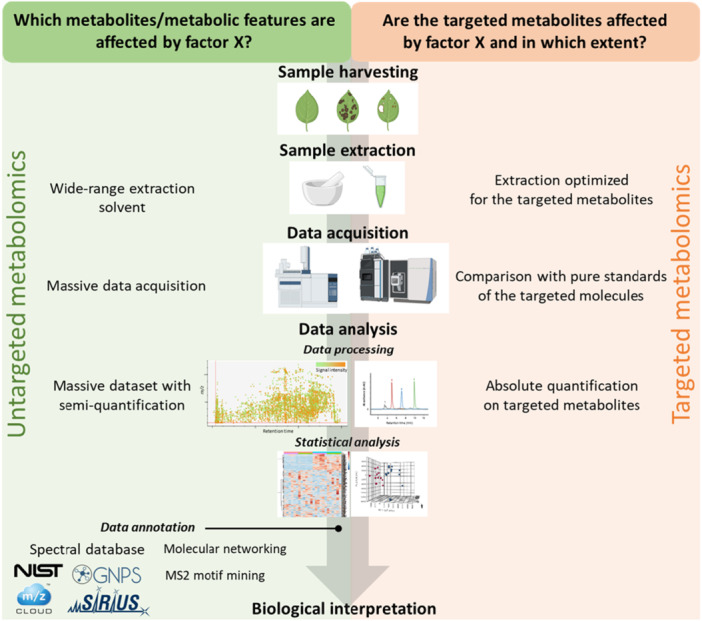
Typical workflow for both untargeted and targeted metabolomics. The choice between targeted or untargeted approaches depends on the scientific question. Each approach comprises four steps. With the exception of sample harvesting, each step has an approach‐specific protocol. The data annotation step is specific to the untargeted approach and is considered very challenging (Li and Gaquerel 2021; Perez de Souza and Fernie 2024). This involves comparing the MS^2^ profiles to public or in‐house spectral databases. As the diversity of spectral databases do not cover the structural diversity of plant metabolism, computational approaches have led to the development of complementary tools to assist with metabolite annotation from MS² data. For example (i) in silico fragmentation tools can extend the available fragmentation data from known metabolites, against which, unknown compounds can be compared (Wolf et al. 2010); (ii) molecular networks can classify unknown metabolites based on partial MS² profile similarity with known metabolites (Allard et al. 2016; Olivon et al. 2017; Nothias et al. 2020), and (iii) fragmentation spectra can be mined for specific fragmentation motifs that reveal substructures helping annotation of unknown compounds or, at least, classification into metabolic families (Dührkop et al. 2019; Rogers et al. 2019). Although plant metabolome annotation remains a major bottleneck, the dynamic of research in this domain, supported by artificial intelligence, could lead to major breakthroughs in the coming years as it has been achieved recently with the emergence of the AlphaFold software for the protein 3D structure elucidation (Jumper et al. 2021; Varadi et al. 2024). [Color figure can be viewed at wileyonlinelibrary.com]

Advances in analytical technologies over the past two decades have significantly expanded access to the plant metabolome. Core tools such as liquid/gas chromatography coupled with mass spectrometry (LC/GC‐MS) and nuclear magnetic resonance (NMR) are now widely available for metabolomic studies. In parallel, robust analytical metabolomic workflows have been developed (Figure 2). Such developments have facilitated the emergence of comparative metabolomics to elucidate plant‐pathogen and plant‐herbivore interactions. The main objective is to compare the accumulation of metabolites in plant tissues in order to understand defence mechanisms. A common approach is to analyze genotypes with contrasting resistance to pests or pathogens in order to identify biomarkers for breeding. This strategy has revealed resistance‐related metabolites in crops such as tomatoes, cocoa, *Medicago truncatula*, *Senecio* hybrids, and chrysanthemums (Leiss et al. 2009, 2013; Mirnezhad et al. 2010; Cai et al. 2017; Knollenberg et al. 2020). An alternative strategy is to target metabolites known to be induced after pest/pathogen attack, including terpenoids, alkaloids, and phenolics with deterrent or toxic effects (Kliebenstein et al. 2002; Agrawal and Kurashige 2003; Nanda et al. 2021). The identified metabolites vary widely across species and pathosystems, being influenced by factors such as developmental stage, environmental conditions and biotic interactions (Badri et al. 2013; Mansfield et al. 2017; Macel et al. 2019; Farahbakhsh et al. 2019; Seybold et al. 2020; Mansfeld et al. 2020; Christensen et al. 2021; Zhang et al. 2021). Metabolomics also provides insight into the timing and spatial distribution of plant defences. However, to confirm a biomarker's role in resistance, functional validation is essential. While comparative metabolomics successfully identifies resistance‐associated metabolites, it provides limited information about their genetic control and heritability. To integrate these metabolic insights into breeding programmes, approaches combining metabolic profiling with genetic mapping are essential. This integration represents the next frontier in understanding resistance mechanisms.

### 1.3 Combining Quantitative Genetics With Metabolomics to Dissect Plant Resistance Mechanisms

As previously detailed in this review, both QTL mapping and metabolomic studies independently exhibit limitations to narrow down the number of candidate genes and metabolites associated with plant resistance. Despite this partial knowledge, breeders can still use these insights to create and select resistant varieties. However, to effectively combine diversified molecular mechanisms within an optimal genotype, it is essential to fully characterise the functions of causal genes and the mode of action of key metabolites. In this last section, we will review studies that focus on dissecting the genetic control of plant metabolism by searching for co‐localisations between genomic regions associated with resistance to pests/pathogens and metabolite‐related loci. Finally, we will discuss the advantages and limitations of this methodology in the context of its application in plant resistance breeding.

**Figure 3:**
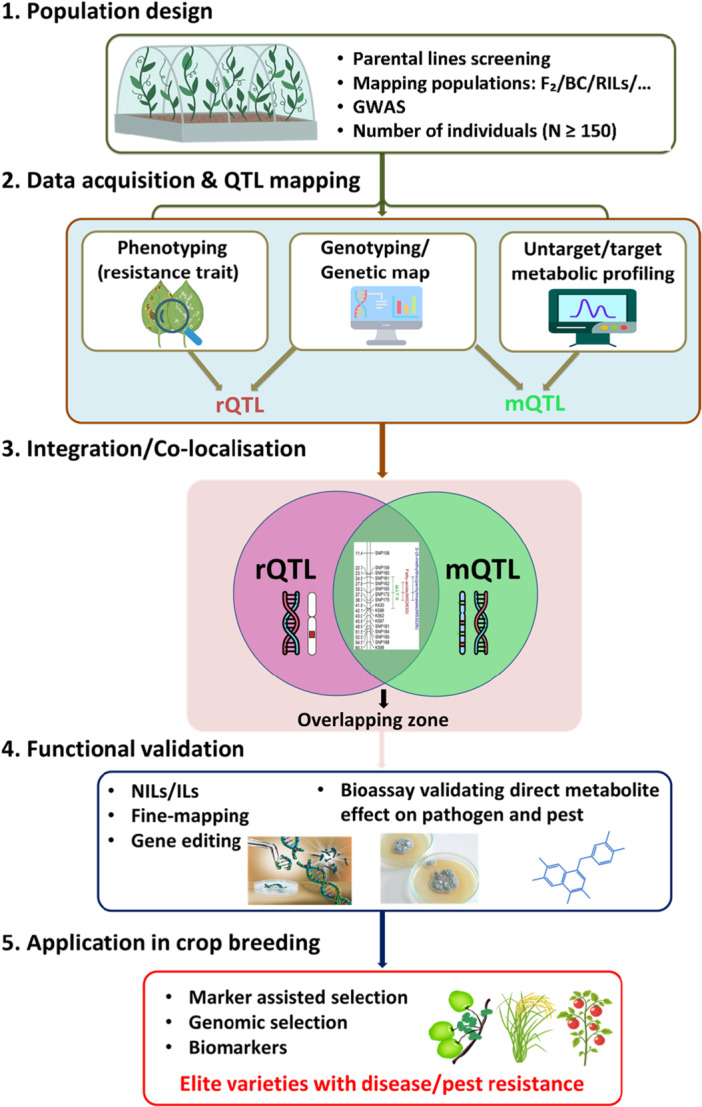
Integrating resistance and metabolic QTL mapping for crop breeding. QTL mapping studies rely on genetic populations (summarised in Box 1 and in the above section of the manuscript) on which experiments are conducted. By combining genotyping data with (i) resistance phenotyping and (ii) metabolomics, researchers can map resistance QTL (rQTL) and metabolic QTL (mQTL), respectively. Then, the genetic positions of these QTLs can be compared directly to identify genomic co‐localisations (where confidence intervals overlap). Identifying such overlapping loci can narrow down candidate genes and metabolites that may be involved in resistance. The involvement of these candidates should be confirmed through complementary studies such as gene editing for candidate genes or bioassays for metabolite candidates before being incorporated into elite material through breeding schemes. [Color figure can be viewed at wileyonlinelibrary.com]

1. **Emerging studies linking genetic control of both specialised metabolism and resistance‐related phenotypic traits**
  The integration of metabolic profiling with QTL analysis has transformed plant metabolome research by identifying genomic regions linked to metabolite variations, thereby revealing loci or genes involved in essential biosynthetic pathways (Carreno‐Quintero et al. 2012; Ding et al. 2020). The co‐localisation of mQTLs and phenotypic QTLs provides crucial insights into the genetic and biochemical mechanisms underlying complex traits (Figure 3). In particular, this co‐localisation might help to understand how metabolic variation can shape observable phenotypes. This approach has been widely applied to the study of various agronomic traits, including fruit quality, yield, and resistance to abiotic stress (Schauer et al. 2006; Hill et al. 2013, 2015; Shi et al. 2020; Labadie et al. 2020). For instance, the co‐localisation of mQTLs from the flavonoid‐lignin pathway with phenotypic QTL influencing plant height suggests a role for specialised metabolites in regulating the growth of foxtail millet (Wei et al. 2023). Likewise, the association between mQTLs for carotenoids and QTLs for soluble solids underscores the putative role of carotenoids in tomato fruit quality and nutritional value (Capel et al. 2015). In the context of water stress, the co‐localisation of a mQTL for metabolites from the tricarboxylic acid cycle, and a QTL for grain yield and harvest index, suggests a link between energy metabolism and drought adaptation in bread wheat (Hill et al. 2013). Beyond agronomic‐related traits, extending this perspective to resistance traits offers a powerful approach to decipher how metabolic variations contribute to plant defence mechanisms.
  To date, around 10 studies have applied this approach in plant—insect and—pathogen interactions (Table 1). Interestingly, the advances of high‐throughput sequencing technology combined with metabolomic tools improvement offered the opportunity for most species to employ the mQTL/rQTL approach, even those with limited genomic information (Kuzina et al. 2011; Feiner et al. 2021). As far as we know, this method has not yet been applied to major crop species such as rice, wheat or maize. For these species, which benefit from extensive genetic and genomic resources, it appears that alternative methods, such as construction of near‐isogenic lines (NILs, see Box 1) (Hamzehzarghani et al. 2008; Gunnaiah et al. 2012; Rossouw et al. 2019) or fine‐mapping populations, have been preferred to characterise molecular mechanisms underlying quantitative resistance. Nonetheless, the mQTL/rQTL approach has been used on tomato and on the model species *Arabidopsis thaliana* (see Table 1).
2. **Searching for mQTL/rQTL co‐localisations highlights specific features of genetic resistance**
  The research of co‐localisations between mQTLs and rQTLs reveals particular patterns in the genetic architecture of plant metabolism. Specifically, ‘mQTL hotspots’ were recurrently found and referred to a large number of mQTLs—between 10 and 200—clustering in one specific genomic region (van den Oever‐van den Elsen et al. 2016; Vosman et al. 2019; Maharijaya et al. 2019; Wagner et al. 2019; Koutouan et al. 2023). Interestingly, the detection of mQTL hotspots has been attributed to different molecular mechanisms: for instance in tomato, Vosman et al. (2019) identified a major hotspot of mQTLs co‐localising with a rQTL and a QTL for type‐IV trichome density. They proposed that the gene underlying such pattern may play a role in regulating type‐IV trichome development, where acylsugars are synthesised and stored. On the other hand, on rapeseed, Wagner et al. (2019) found a mQTL hotspot co‐localising with a major‐effect rQTL but also with the R gene *Pb‐Bn2*. Since some rQTL are thought to be altered form of R genes, often implicated in recognition of pests/pathogens, the co‐localisation of a mQTL hotspot with a major‐effect rQTL could suggest that the causal gene may encode a receptor that triggers a metabolic cascade.
  Alternatively, other rQTLs may have a lower effect and less direct impact on metabolites. The underlying genes could be either involved in signalling regulation or enzyme production of a biosynthetic pathway. For instance, Rowe and Kliebenstein (2008) identified camalexin‐QTL co‐localising with a rQTL but no camalexin biosynthetic genes were found in the underlying genomic region. Yet, they proposed the involvement of the rQTL in camalexin signalling rather than in its synthesis. In carrot, Koutouan et al. (2023) identified around 10 mQTLs for terpenes co‐localising with rQTL. Intriguingly, a fraction of these mQTLs shared similar levels of explained phenotypic variance with associated rQTLs, suggesting terpenes as the most evident candidates behind *Alternaria dauci* resistance. Alternatively, lower explained phenotypic values in mQTLs would suggest a ‘cocktail effect’ where several different metabolites are needed to achieve observed levels of resistance (Maharijaya et al. 2019). Moreover, transcription factor and terpene synthase genes were highlighted as good candidates in the overlapping confidence intervals of rQTLs leading to further transcriptomic analyses (Koutouan et al. 2023). In this study, functional analyses including bioassays revealed that two of the seven candidate terpenes exhibited antifungal activity, suggesting a direct role for these compounds in disease resistance to *A. dauci*. Therefore, studying simultaneously the genetic control of specialised metabolism and resistance can shed light on the molecular mechanisms underlying rQTLs whether they have a role in recognition, signalisation or defence compound production.
3. **Challenges and future directions**

**Table 1:** Summary of studies employing the mQTL‐rQTL approach.

Crop	Biotic stress		Metabolomic analyses	Phenotypic trait studied	Range of phenotypic variance explained by resistance QTL (%)	Putative or presumed mechanisms identified	Exploration of candidates		Reference
	Type	Precisions					Genes	Metabolites	
*Arabidopsis thaliana*	Pathogen	*Botrytis cinerea* (fungus): tw*elve individual isolates*	Targeted on camalexin	Measures of fungal lesion diameter	2–12	Camalexin signalling	—	Antimicrobial role of camalexin already known	Rowe and Kliebenstein (2008)
Wintercress (*Barbarea vulgaris*)	Pest	*Phyllotreta nemorum* (herbivore)	Targeted LCMS on saponins and glucosinolates	Larvae survival and leaf hairiness	24–30	Identification of two resistance QTL probably linked to saponin production. No link between hairiness, glucosinolate and these resistance loci.	—	Already known inhibitory feeding effect for one saponin out of the four.	Kuzina et al. (2011)
Wild tomato *(Solanum galapagense)*	Pest	*Bemisia tabaci* (whitefly): one specie	Untargeted GCMS and targeted LCMS for acylsugars in bulks	Pre‐adult and adult survival, oviposition rate, number and trichome type	9–80	Regulation of trichome Type IV formation, where specific metabolites including acylsugars would be synthetised and stored	—	—	Firdaus et al. (2013)
Turnip *(Brassica rapa)*	Pathogen	*Botrytis cinerea* (fungus): five individual isolates	Targeted on glucosinolates	Measures of lesion size	Not explicit: minor	—	—	Depending of the pathogen/pest, GSL can have toxic properties or alter defence signalling pathways	Zhang et al. 2016
Wild tomato (*Solanum pennellii*)	Pest	*Bemisia tabaci* (whitefly): one specie	Untargeted GCMS	Adult survival and oviposition rate	Around 10	Identification of metabolite candidates (including precursors of acylsugars) that need futher bioassays			van den Oever‐van den Elsen et al. (2016)
Pepper *(Capsicum sp.)*	Pest	*Frankliniella occidentalis* (thrips): one specie	Untargeted LCMS	Larvae survival and leaf damage	44–52	Identification of candidates (diterpene glycoside and capsianosides) that need further bioassays	—	—	Maharijaya et al. (2019)
Grapevine (*Vitis sp*.)	Pathogen	*Plasmopara viticola* (oomycete)	Targeted on phenolics especially stilbenoids	Disease severity and incidence measurements on inoculated plants and on detached leaves	10–20	—	RT‐qPCR	—	Vezzulli et al. (2019)
Wild tomato *(Solanum galapagense)*	Pest	*Bemisia tabaci* (whitefly): two species	Untargeted LCMS and targeted LCMS for acylsugars	Trichome density, number of living whiteflies and eggs	2–80	Regulation of trichome Type IV formation, where specific metabolites including acylsugars would be synthetised and stored	—	—	Vosman et al. (2019)
Rapeseed (*Brassica napus*)	Pathogen	*Plasmodiophora brassicae* (protist)	Untargeted LCMS and targeted of non‐structural carbohydrates, polyols, organic acids, and amino acids	DNA pathogen quantification and Disease Index	1–80	Constitutive accumulation of four compounds may play a role in partial resistance, specifically citric acid could represent a consistent source of organic carbon for the pathogen	—	—	Wagner et al. (2019)
Hop (*Humulus lupus*)	Pathogen	*Pseudoperonospora humuli* (oomycete)	Untargeted LCMS with 45 standards known to be present in hops	Percentage of fungal sporulation on leaves	Not explicit	Phenylpropanoids as prophylactic compounds (constitutive) with a direct activity or as precursors of active compounds	—	Two phenylpropanoids were tested for their protective activity *in planta*, in cocktail	Feiner et al. (2021)
Carrot (*Daucus carotta*)	Pathogen	*Alternaria dauci* (fungus): natural infestation	Targeted on terpenes	Percentage of foliar damages	7–20	Two terpene candidates out of seven showed antifungic activty suggesting a direct role in resistance	Microarray analysis	In vitro bioassays for four terpenes	Koutouan et al. (2023)

Abbreviations: GCMS and LCMS, gas and liquid chromatography coupled to mass spectrometry; QTL, quantitative trait loci; RT‐qPCR, real‐time quantitative polymerase chain reaction.

The combined mQTL/rQTL approach relies on the hypothesis that resistance is based on specialised metabolism although other types of defence‐related molecules, such as pathogenesis‐related (PR) proteins, may also play an important role. In addition, these approaches are inherently limited by the subset of metabolites that can be detected, which depends on the extraction methods, solvents, and analytical settings employed. Consequently, a lack of co‐localisation between mQTLs and rQTLs, as observed by Vezzulli et al. (2019), does not represent an absence of information. Rather, it provides valuable insights by suggesting that the resistance mechanism underlying the rQTL may not be directly associated with the specialised metabolites detected, and it invites further exploration of alternative explanations—ranging from experimental and technical biases to genuinely distinct defence pathways.

To take this further, additional resources may be useful for characterising the molecular functions of rQTLs and complement this approach. For instance, other omics datasets such as whole genomes (Kuzina et al. 2011; Vezzulli et al. 2019) or transcriptome could be used to explore candidate genes (e.g., targeted expression analyses in Vezzulli et al. 2019; Koutouan et al. 2023, or untargeted RNA‐seq study in van Haperen et al. 2021). The approach is indeed transferable to transcriptomic datasets, which can also be combined with QTL mapping to identify expression QTLs (eQTLs), as well as proteomic datasets for protein QTLs (pQTLs) (Cubillos et al. 2012; Zhou et al. 2021). Ultimately, combining QTL mapping with molecular datasets and looking for multiple QTL co‐localisations in ‘system genetic approaches' will help researchers to unravel plant defence mechanisms (Clark et al. 2024).

One of the benefits of the approach is to narrow down the number of candidate genes and metabolites in comparison to linkage mapping and comparative metabolomic studies. The list of candidate genes can further be reduced by focusing, primarily, on the overlapping part of mQTL and rQTL intervals (Vosman et al. 2019; Koutouan et al. 2023). The resolution of rQTL intervals remains crucial because broad intervals limit the power of colocalization, whereas fine mapping and the identification of recombinant individuals can narrow these intervals, guide targeted re‐metabotyping, and improve mQTL precision. MetaQTL mapping offers complementary path to the search for co‐localisations, weaving together multiple datasets and genetic backgrounds in broader meta‐analyses (Badji et al. 2018; Wagner et al. 2019). Finally, while gene annotation continues to advance steadily, metabolite annotation remains laborious, and current databases still require further enrichment.

Following the identification of candidates, a final step of validation is still needed for both genes and metabolites. Gene validation remains challenging for some species because transformation or regeneration of cells, tissues, or plants is often time‐consuming and labour‐intensive, requiring specialised experimental skills. In addition, producing transgenic plants for specific genotypes remains complex, and the biological basis for genotype‐dependent transformation is still largely unexplained (Anjanappa and Gruissem 2021). For metabolites, about half of the studies in this field employ untargeted approaches, which allow the discovery of novel metabolites, but suffered from limitations in precisely quantifying variation in metabolic content (Table 1). Thus, additional targeted metabolic analysis is often required to accurately assessed quantitative differences in candidate metabolite contents and explore their role using in vitro bioassays (Firdaus et al. 2013; Koutouan et al. 2023). Although precise quantification remains challenging, recent methods using labelled isotopes (Boutet‐Mercey et al. 2018) offer promising alternatives.

Once the number of candidates has been reduced, validating specific compounds with their proposed mode of action in plant resistance requires considerable effort. For example, Koutouan et al. (2023) identified seven candidate terpenes that could explain resistance to *A. dauci*. They tested four of these terpenes for antifungal properties and validated two of them. These results clearly demonstrate that one chemical family can be induced in the plant defence response, while only a few compounds are directly involved in this response. However, it is important to consider the potential ‘cocktail effect’, whereby each component may have limited efficacy against the pathogen individually, yet collectively contribute substantially to resistance. Moreover, thousands of mQTLs can be mapped all over the genome leading to fortuitous co‐localisations and increasing the number of bioassays to be performed. Thus, functional validation remains an essential step, even if time‐consuming. In addition to laboratory assays, the potential protection effect of metabolites can also be evaluated *in planta* by external application (Feiner et al. 2021). Interestingly, the identification of metabolites with external protective roles could motivate the development of eco‐friendly biopesticides (Lavoir et al. 2022) while those acting inside the plant could be the target of breeding schemes, if they have no impact on customer expectations (Le Clerc et al. 2019). Finally, additional studies are needed to explore the variability of metabolite genetic architecture in different plant organs (Kittipol et al. 2019). In order to deploy such findings in commercial varieties, it is important to take into account the possible negative effect in plant organs that are actually consumed.

## 2 Conclusion

The combination of rQTLs and mQTLs in plant‐pest/pathogen interactions studies offers valuable insights into the exploration of biochemical basis of resistance in many species. By identifying loci responsible for resistance trait variation and their co‐localisation with mQTLs, researchers can narrow down the number of metabolites requiring further analyses and experimental validation. New hypotheses can be formulated on the potential genes underlying rQTLs thanks to the genetic architecture of mQTLs and their annotation, if available. These results help unravelling metabolic pathways involved in plant resistance to pests/diseases, which is crucial to propose genetic factors mobilising complementary molecular mechanisms to combine within elite crops by gene pyramiding. This strategy offers concrete prospects for sustainable agriculture by developing durably‐resistant varieties, better adapted to diverse agro‐environmental conditions. Several questions still need to be addressed in order to integrate these results in breeding schemes. For instance, the functions and impacts of identified resistance metabolites could also be investigated to target multi‐pest regulation by testing other pathosystems. Their compatibility with customer expectations should also be considered. Ultimately, this approach provides key elements for ideal chemotype constitution (i.e., genotypes having differences in quantity and quality of their biochemical components). By characterising the genetic control of specialised metabolism, it also informs on which breeding tools (genomic selection, fast track breeding, New Breeding Techniques) would be the most appropriate to quickly and precisely incorporate genetic factors within a potential new variety.

## Conflicts of Interest

The authors declare no conflicts of interest.

## Data Availability

Data sharing not applicable to this article as no datasets were generated or analysed during the current study.
